# Multifunctional Smart Conducting Polymers–Silver Nanocomposites-Modified Biocellulose Fibers for Innovative Food Packaging Applications

**DOI:** 10.1021/acs.iecr.2c01327

**Published:** 2022-08-24

**Authors:** Abdelqader El Guerraf, Sana Ben Jadi, Imane Ziani, Mohammed Dalli, Farooq Sher, Mohammed Bazzaoui, El Arbi Bazzaoui

**Affiliations:** † Laboratory of Applied Chemistry and Environment, Faculty of Sciences, Mohammed First University, Oujda 60000, Morocco; ‡ Laboratory of Materials and Environmental, Faculty of Sciences, Ibn Zohr University, 885 Agadir 80000, Morocco; § Physical Chemistry of Natural Substances and Process Research Team, Laboratory of Applied Chemistry and Environment, Faculty of Sciences, Mohammed First University, Oujda 60000, Morocco; ∥ Laboratory of Bioresources, Biotechnology, Ethnopharmacology and Health, Faculty of Sciences, Mohammed First University, Oujda 60000, Morocco; ⊥ Department of Engineering, School of Science and Technology, Nottingham Trent University, Nottingham NG11 8NS, United Kingdom

## Abstract

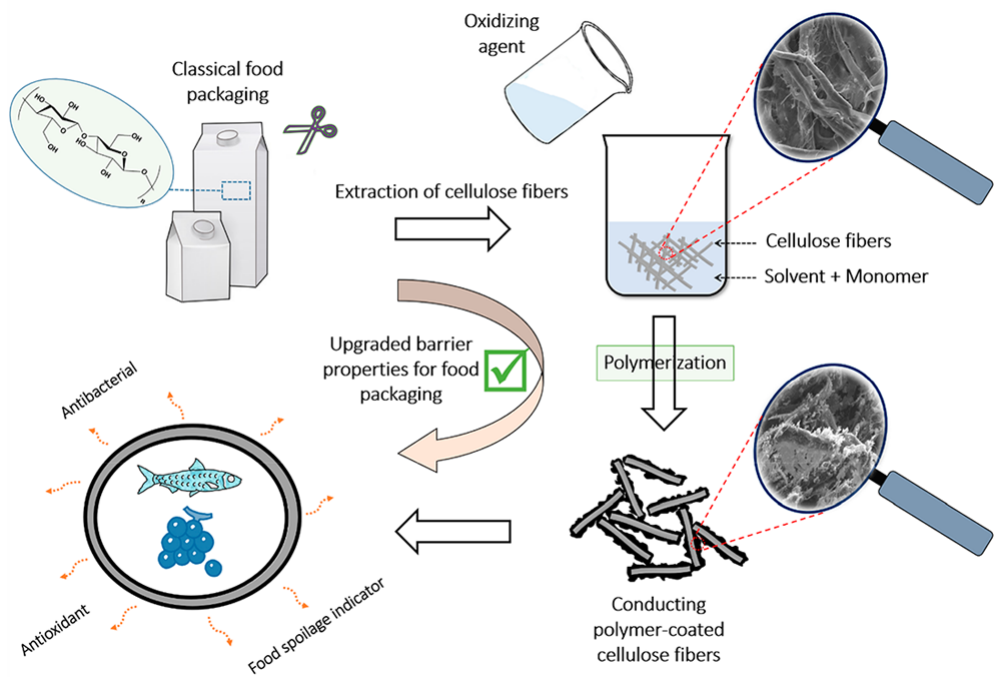

In recent decades, food-packaging markets have attracted researchers’ interest in many ways because such industries can directly affect human health. In this framework, the present study emphasizes the interesting and smart properties provided by new nanocomposites based on conducting polymers (CPs), silver nanoparticles (AgNPs), and cellulose fibers (CFs) and their possible applications as active food packaging. Polyaniline and poly(3,4-ethylenedioxythiophene) containing AgNPs were elaborated on via a simple one-step *in situ* chemical oxidative polymerization on CFs. Spectroscopic and microscopic characterization allowed a full discussion of the morphology and chemical structure of the nanocomposites and confirmed the successful polymerization of the monomer as well as the incorporation of AgNPs into the CP-based formulation. This study aims to demonstrate that it is possible to produce a highly efficient package with enhanced protective properties. Thus, the synthesized nanocomposites were tested as volatile organic compounds, sensors, and antibacterial and antioxidant agents. It is shown that the elaborated materials can, on the one hand, inhibit the development of biofilms and decrease the oxidation reaction rate of foodstuffs and, on the other hand, detect toxic gases generated by spoiled food. The presented method has unlocked massive opportunities for using such formulations as an interesting alternative for classical food containers. The smart and novel properties offered by the synthesized composites can be operated for future industrial applications to prevent any degradation of the packaged products by offering optimum protection and creating an atmosphere that can extend the shelf life of foodstuffs.

## 1 Introduction

In daily life, several metallic and nonmetallic materials are used in the food and pharmaceutical industries. With numerous practical functionalities and interesting characteristics such as durability, gas barrier properties, and freedom of design, materials like tinplate, aluminum, and high-density polyethylene or polycarbonate are widely encountered in the packaging markets and are highly efficient as food containers. They are considered a crucial section of the food industry because they can directly affect human health. Thus, because the latter is one of the global subjects of interest to scientific researchers, food packaging is expected to be stable under various conditions from its manufacturing to the consumption of food by the consumer and must offer optimum protection of the packaged product during this process. During the past decades, classical packaging has demonstrated various problems and has created a large gap in the food industry.

In fact, one of the great concerns nowadays is the use of plastics in the food markets because such materials are not biodegradable and every piece of plastic ever made is still on this planet,^1^ thus generating notable waste pollution and impacting the earth’s ecosystems. In addition, corrosion phenomena can highly limit the usage of metals like tinplate or aluminum as packaging for food despite offering numerous required properties that provide a safe and long shelf-life of products (heat resistance, full recyclability, durability, and good conductivity).^2^ In the presence of specific environments where metallic food containers can be exposed in the food industries (acidic food like fruits and vegetables) and despite the protective layer that usually covers the metals, dissolution of the packaging is always an unavoidable phenomenon. By releasing metallic ions or complexes, corrosion leads to the alteration of organoleptic properties of foodstuffs, which directly affects the quality of the latter and increases the risk to human health.^3^

The growing demand for high-quality food products, long shelf-lives, and low-cost materials has catalyzed the expansion of new processing technologies and new packaging materials. In this context, cellulose-based materials have proven to be very promising as food contact matrixes, especially with their well-known biodegradable properties and nontoxicity. Cellulosic materials have taken a substantial place in our lives, and their use covers a wide range of fields, from bone tissue engineering^4^ to supercapacitors^5^ to biosensors^6^ to wastewater treatment^7^ to applications in pharmaceutical industries.^8^ However, their weak mechanical properties and susceptibility to humidity are significant drawbacks, which led the scientific community to increase its interest in such materials.^1^ In order to satisfy the consumers and society, emerging trends in active packaging systems are highly demanded. Active or smart packaging is expected to do more than the usual classical role (transportation of packaged products from production to places of consumption). They must be capable of interacting with their environment and detecting any disturbance of the system for which they are designed.

Such materials are needed to prevent potential health risks involving human beings, to ensure that the natural characteristic and appearance of the foodstuffs are not radically transformed, and to extend the shelf-life of the packaged products by constituting a safety barrier between the food and the outside environment (inhibiting microorganism development, preventing the migration of contaminants, etc.). In this context, numerous investigations were dedicated to the development of new efficient and active containers. The reported studies concerned the use of essential oils,^9^ natural polymer-based films,^10^ or the incorporation of antimicrobial nanoparticles (NPs) within packaging materials.^11^ Some other works focused on the modification of cellulose-based materials to enhance their physicochemical properties. Lavoine et al.^12^ have succeeded in the preparation of a novel bactericidal biomaterial by grafting cyclodextrin onto microfibrillated cellulose.

Additionally, antibodies to bacteria lysozyme and lactoferrin were added to paper containing (carboxymethyl)cellulose.^13^ The synergistic effect between the two simultaneously released proteins was demonstrated in examinations against common food contaminants. de Moura et al.^14^ have discussed the elaboration of cellulose-based antimicrobial nanomaterial with incorporated silver nanoparticles (AgNPs) and their use as effective food systems. The present research is directed toward the use of electronically conducting polymers (CPs) in the food industry. The use of CPs in such industries is highly limited. Only a few attempts have been reported and concern mainly polypyrrole (PPy)-coated cellulose paper,^15^ PPy and polyaniline (PAni) combined with cotton fabric,^16^ and PPy/nanocellulose composites.^17^ This class of organic materials has shown remarkable physicochemical properties, leading to countless applications in various fields.^18^ With high stability, a simple synthesis method, and nonsolubility in common solvents, CPs are considered to be a novel generation of nanomaterials. Such polymers are also recognized by their electroactivity, which allows them to be doped with various species. Because metallic NPs such as AgNPs are highly active as antimicrobial agents and have gained considerable interest as a result of their action toward an extensive spectrum of microorganisms and fungi,^19^ their incorporation into CPs matrixes can offer good preservation of food quality by slowing down or preventing microbial growth and provides other functional attributes such as use as an antioxidant and a gas sensor.^19^ The present work proposes a novel framework in which a synergistic effect can be employed between CPs and AgNPs, resulting in active food packaging with improved bactericidal and scavenging characteristics.^16^

On the basis of the literature review presented above, different biomaterials were used in food-packaging industries. Several researches were dedicated to the improvement of classical food containers to extend the shelf-life of packaged products. Despite the interesting properties offered by the described biomaterials, there is always a great need for novel food packaging. In addition, the researcher’s biggest concern is to find an economic and environmental process for elaboration of the desired material with enhanced protective properties. Thus, the primary objective of the current study is to fulfill this great gap demonstrated by the food market and to elaborate on, by a simple one-step synthesis, new and effective food-packaging materials endowed with advanced smart properties. The primary focus is on the use of novel nanocomposites based on CPs, AgNPs, and cellulose. Unlike the usual biomaterials, in the present work, the biodegradable property of the cellulosic matrix is combined with the bactericidal performance offered by AgNPs as well as the smart characteristic of CPs to prepare a container that offers optimum protection of the packaged products. In a first step, the *in situ* oxidative polymerization of aniline (Ani) and 3,4-ethylenedioxythiophene (EDOT) was achieved in the existence of cellulose fibers (CFs). Afterward, the coated cellulose was treated with a silver nitrate solution to obtain silver-based nanocomposites. The chemical and morphological characterization was examined using X-ray diffraction (XRD), Fourier transform infrared spectroscopy (FTIR), and scanning electron microscopy (SEM). The elaborated coatings were then evaluated for their sensing characteristics toward volatile organic compounds (VOCs) as well as for their antibacterial and antioxidant activities.

## 2 Experimental Section

### 2.1 Synthesis of PAni and Poly(3,4-ethylenedioxythiophene) (PEDOT) Coatings on CFs

Elaboration of the CP-based nanocomposites was achieved with a simple one-pot *in situ* synthesis. In the case of PAni, polymerization was done in an acidic medium to enhance the solubility of the monomer.^20^ First, 0.5 g of the CFs (extracted from real Tetra Pak food packaging) was maintained under stirring for 30 min in a solution of 1 M hydrochloric acid (HCl) and 0.2 M Ani (≥99.5%, Sigma-Aldrich). This process ensured adsorption of the monomer along the length of the fibers and increased full coverage and adherence of the resulting polymer. Subsequently, an acidic solution of 0.2 M potassium persulfate (K_2_S_2_O_8_, Sigma-Aldrich) was added dropwise to this solution, and the mixture instantly became green, indicating the start of polymerization. The reaction was left for 24 h under regular magnetic stirring, and the PAni composite was carefully cleaned with 1 M HCl to eliminate all residues and dried at 80 °C. The synthesis of PAni using K_2_S_2_O_8_ follows eq 1:^21^
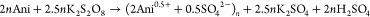
1

PAni is 50% doped with SO_4_^2–^, which corresponds to a partial positive charge of 0.5+ on each nucleus of the polymer backbone. K_2_S_2_O_8_ involves two electrons (eq 2), and the extraction of two electrons is required for the coupling of each Ani unit. Hence, a [K_2_S_2_O_8_]/[Ani] molar ratio of about 1.25 is generally used.^21^
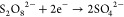
2

PEDOT was produced on CFs following the same procedure as PAni. In this case and with the poor solubility of the EDOT monomer in aqueous media known, polymerization was realized in a methanol solution. Like the previous case, 0.5 g of cellulose was mixed with 0.2 M EDOT (98%, Sigma-Aldrich) in a solution of methanol (Sigma-Aldrich) and left to stir for 30 min. Polymerization began with the addition of 0.2 M copper chloride (CuCl_2_, Sigma-Aldrich), the solution became black after a few minutes, and the reaction was left for 20 h. The covered fibers were removed from the reaction mixture, rinsed with purified water and methanol, and dried at 90 °C. The polymerization reaction of EDOT is as follows (eq 3):^22^
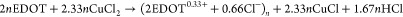
3

**Figure 1:**
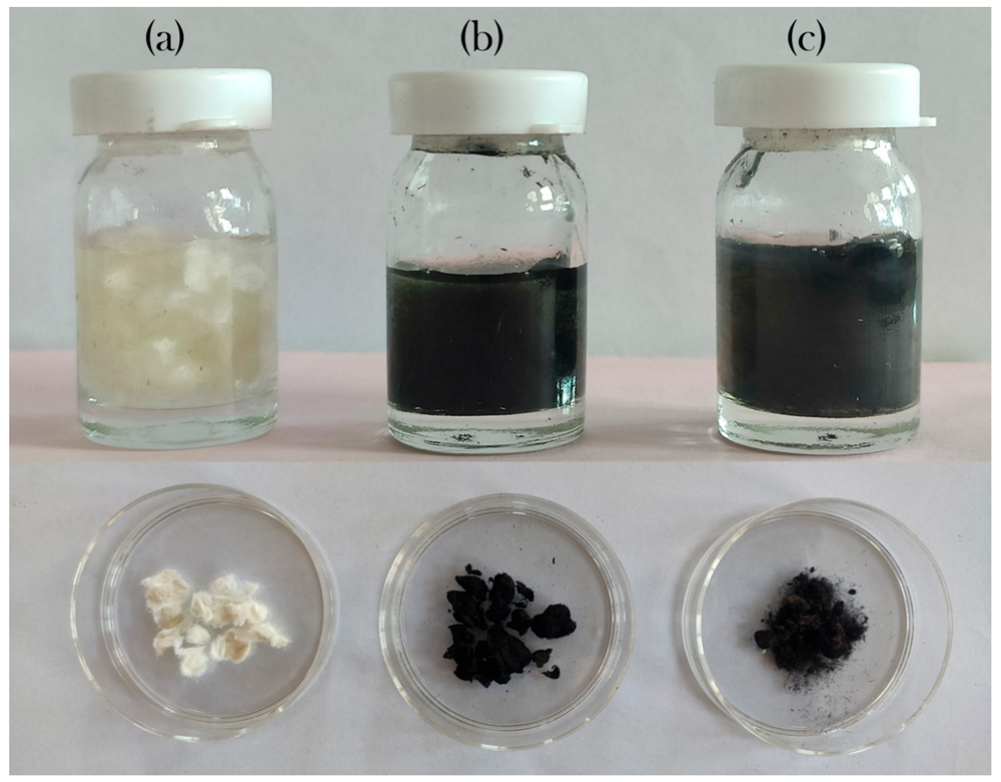
Photograph of bare CFs: (a) PAni-coated CFs; (b) PEDOT-coated CFs; (c) before and after separation from the reaction mixture.

The synthesized PEDOT coating is doped to 33% with the chloride anions from the oxidizing agent. The latter involves two electrons; two electrons are required for the α–α′ coupling of each monomer nucleus and 0.33 electrons for the oxidation of EDOT (i.e., a positive charge on about three EDOT units). Therefore, an oxidant-to-EDOT molar ratio of about 1.16 should be considered. However, a lower amount of oxidant will often be used (a ratio of 1) to avoid overoxidation of the elaborated polymer. The two reactions (eqs 1 and 3) lead to the formation of homogeneous PAni and PEDOT (Figure 1) doped with sulfate and chloride ions, respectively. The color of the elaborated nanocomposites can be monitored by modifying the experimental synthesis conditions (the doping anion, grafting of the monomer with specific components, etc.). However, the original black color obtained after completion of the reaction is very important in the food-packaging industry because it can protect the packaged product from light degradation.

### 2.2 Incorporation of AgNPs

PAni and PEDOT coatings were then used for the reduction of silver cations. This procedure aims to prepare composites containing AgNPs for further use in the packaging market. The protocol consists of immersion of the coated CFs in a 0.1 M silver nitrate (Sigma-Aldrich) aqueous solution. The reaction mixtures were stirred for 24 h under visible light, and then the samples were collected, washed with purified water, and dried at 90 °C. A redox reaction occurred between the unoxidized segments of the polymer and the silver cations, resulting in the formation of a polymer–silver nanocomposite.

### 2.3 Characterization

Initially, the elaborated coatings were subjected to a 100 °C temperature for 48 h. The morphology of bare and coated CFs was observed by SEM (Zeiss EVO 40) in conjunction with energy-dispersive X-ray spectroscopy (EDS; Bruker-QUANTAX). The SEM filament was operated at variable currents and an accelerating voltage of 20 kV using 1.00K× and 10.00K× magnifications. A Jasco FTIR 4700 spectrometer was used to carry out attenuated-total-reflectance Fourier transform infrared (ATR-FTIR) analysis. All absorption spectra were recorded in the range of 500–4000 cm^–1^. The XRD patterns of all composites were recorded on a Shimadzu XRD-6000 diffractometer using a Cu Kα (λ = 0.154 nm) radiation source. The data were recorded with a 0.02°/s scanning speed in a diffraction angle range from 10° to 80°. The crystallite sizes of the AgNPs were calculated using the Scherrer formula (eq 4)^23^
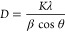
4where *D* (nm) is the size of the silver crystal, *K* is the Scherrer constant (0.90), λ is the radiation wavelength of the X-ray (0.154 nm), β is the half-width height of the diffraction peak (degrees), and θ is the diffraction angle of the associated *hkl* plane. The average size of the silver was determined through the (111) plane.

### 2.4 Equipment for Vapor Sensing

Gas detection experiments were conducted with a homemade apparatus as detailed in previous studies.^15,24^ Briefly, a specific volume of an ammonia (NH_4_OH, Sigma-Aldrich) or acetone (C_3_H_6_O, Sigma-Aldrich) solution was placed in a round-bottom flask, which gives a two-phase (liquid–gas) equilibrium. The synthesized composites were compressed into small pellets of 7 mm diameter and 1 mm thickness and fixed, using a conducting glue, between two copper cables in the analysis chamber. The vapors could be easily transported to this chamber using a stopcock and a circulating fan. A GWINSTEK GDM-8342 digital multimeter was used to record the variation in the electrical resistance of the tested samples during exposure to vapor (gas injection) and clear air (air purging). This change was followed over time, and the response curves were obtained in tabular form.

### 2.5 Bacterial Strains and Agar Diffusion Test

Bacterial strains *Escherichia coli* ATCC 25922 and *Staphylococcus aureus* ATCC 29213 were used to characterize the bactericidal performance of the composites. For this, a well-known method, namely, the agar diffusion test, was adopted. Fresh cultures of bacterial strains were diluted to the physiological solution to prepare bacterial suspensions. The turbidity of the suspensions was normalized to the 0.5 McFarland standard with a nephelometer (Biosan). Afterward, the Petri dish surface was inoculated with the suspensions, and the tested samples, compressed into disks with a 7 mm diameter, were positioned on the Mueller Hinton agar surface. The agar plates were incubated for 20 h at 37 °C, and the zone of inhibition was measured by a caliper. All assays were achieved in triplicate.

### 2.6 Free-Radical Scavenging Assay by 1,1-Diphenyl-2-picrylhydrazyl (DPPH)

The scavenging activity of the CP-based nanocomposites was assessed using a stable DPPH free radical (DPPH^•^) following the protocol described elsewhere with slight modification.^17^ The assay was based on measurement of the ability of the composite to reduce the free radical by giving a hydrogen atom to DPPH^•^, thus becoming paired off (DPPH-H) or converting to a DPPH anion through an electron-transfer reaction.^25^ Briefly, 2500 μL of DPPH in ethanol (0.04 mg/mL) was mixed with different amounts of the nanocomposite (0.2, 0.5, 1, and 2 mg for PAni-based coatings and 1, 5, 10, and 15 mg for PEDOT coatings). The resulting solution was allowed to mix for 30 s and incubated in dark conditions at room temperature. The absorbance was collected every 10 min at 517 nm using a UV–vis spectrophotometer (Shimadzu UV 1650-PC). The scavenging activity (%) of DPPH^•^ radicals was calculated using eq 5:^17^
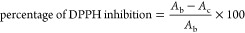
5where *A*_b_ is the absorbance of the blank (DPPH + ethanol) and *A*_c_ is the absorbance of the composite (DPPH + composite + ethanol). Three measurements were conducted for each experiment; the results were reported as the mean value, and the error bars were represented from the standard deviation.

## 3 Results and Discussion

### 3.1 Structural and Morphological Characterization

#### 3.1.1 Crystallographic Analysis

**Figure 2:**
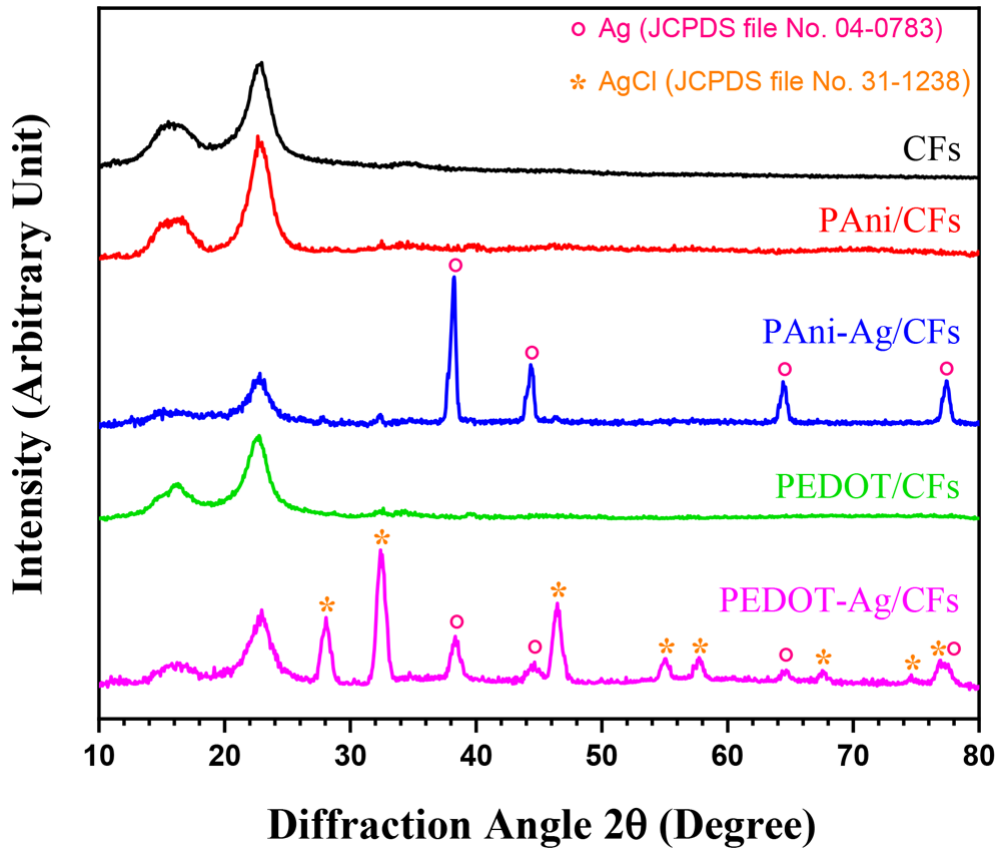
XRD patterns of the coated and uncoated CFs.

The XRD patterns of bare and CP-coated CFs are gathered in Figure 2. The diffractogram recorded for CFs indicates the presence of three diffraction peaks at 14.9°, 16.4°, and 22.8° assigned to the cellulosic crystalline phase, more precisely, the (101), (102), and (200) planes.^26^ Similar peaks were observed after the modification of CFs with PAni and PEDOT. In fact, such types of polymers are recognized by an amorphous nature with a degree of crystallinity lower than 5%.^27^ Commonly, PAni and PEDOT display one broad peak with a lower intensity at 2θ = 20°. It is difficult to see this peak because its overlap with those of cellulose appearing with higher intensities. The XRD pattern of silver-based nanocomposites is shown in the same Figure 2. The PAni-Ag-coated CFs revealed, in addition to the peaks related to the cellulose structure, four other lines with 2θ values of 38.1°, 44.4°, 64.4°, and 77.3° attributed respectively to the (111), (200), (220), and (311) Bragg reflections of the face-centered-cubic phase of silver.^26^ These Bragg reflections are in good agreement with the JCPDS data for metallic silver (JCPDS 04-0783).

Likewise, the PEDOT-Ag coating exhibits all peaks associated with the cellulose and AgNPs. In addition, the diffractogram shows several reflections at 28°, 32.4°, 46.4°,55°, 57.8°, 67.5°, 74.5°, and 76.9°, which correspond to the (111), (200), (220), (311), (222), (400), (311), and (420) planes, respectively.^28^ This crystalline phase reveals the existence of a cubic phase of silver chloride in the composites (JCPDS 31-1238), which was generated by interaction of the Cl^–^ doping anion trapped in the polymer chains with a silver cation during elaboration of the PEDOT-Ag/CFs composite. The average crystalline sizes of AgNPs obtained from the (111) diffraction line using Scherrer’s equation are 15.59 and 9.58 nm for PAni-Ag/CFs and PEDOT-Ag/CFs, respectively.

#### 3.1.2 Vibrational Characterization

**Figure 3:**
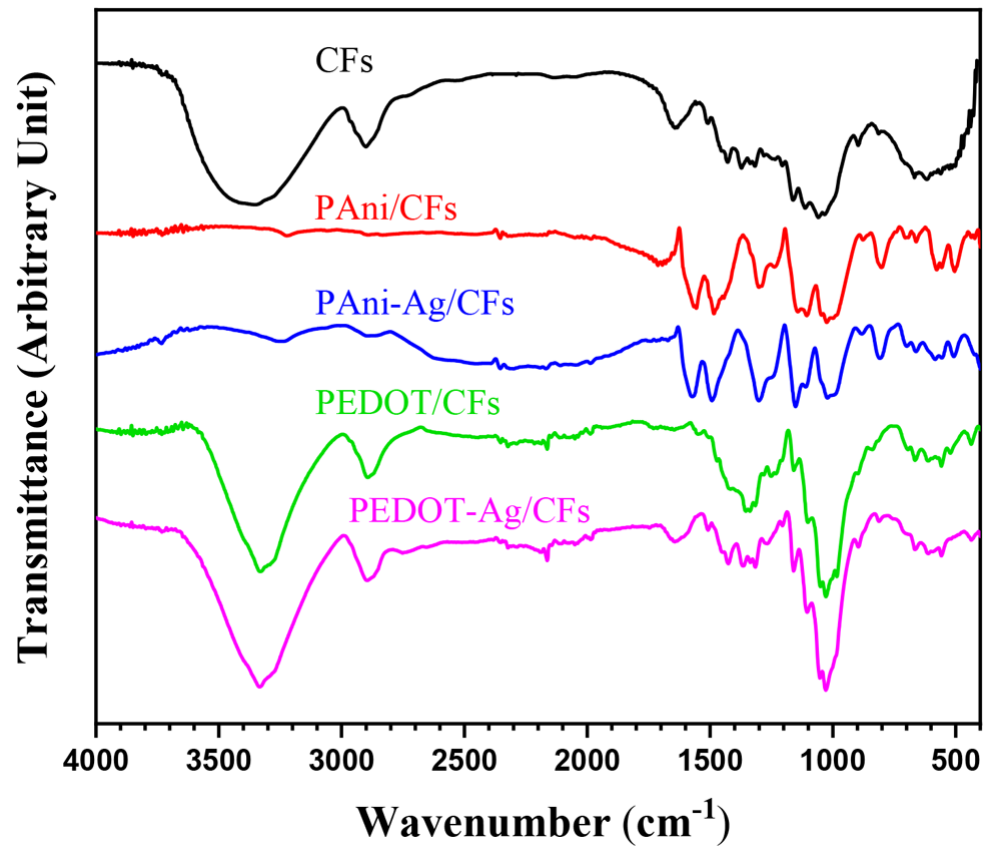
ATR-FTIR spectra of the coated and uncoated CFs.

Vibrational analysis was achieved to gain additional information on the chemical structure of the nanocomposites. First, the FTIR spectrum of CFs (Figure 3) demonstrates the presence of all characteristic peaks of cellulose. The broad peak at 3363 cm^–1^ is attributed to the O–H stretching vibrations.^17^ The stretching of the C–H and C–C bonds is confirmed by the band at 2902 cm^–1^.^29^ The peak around 1643 cm^–1^ characterizes the in-plane vibration of the absorbed water H–O–H, and the one observed at 1432 cm^–1^ results from the in-plane deformation of C–O–H at C6.^30,31^ The bands between 1200 and 1400 cm^–1^ are associated with the H–C–H wagging and rocking with C–C–H,^32^ and those situated at 1165 and 1063 cm^–1^ correspond to the stretching of C–O–C and C–C–O, respectively.^33^ The C–H stretching vibrations for β-glycosidic in different groups of lignin, cellulose, and hemicellulose are detected at 1111 and 896 cm^–1^,^34,35^ and the bands at 667 and 618 cm^–1^ are assigned to the out-of-plane vibrations of the O–H bonds.^15^

**Figure 4:**
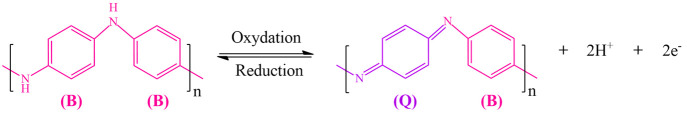
Transition between benzenoid B and quinoid Q structures during the doping–dedoping process of PAni.

The spectra obtained for PAni before and after the introduction of AgNPs are almost identical. A low-intensity peak observed at about 3223 cm^–1^ may result from the stretching vibration of the N–H group, which suggests that the structure of the coating contains both primary and secondary amines.^36^ Two intense bands at 1561 and 1484 cm^–1^ are linked to the C=C stretching vibrations of the quinoid (Q) and benzenoid (B) rings, respectively (Figure 4). The presence of these Q and B bands indicates that PAni is composed of imine and amine units.^37^ The stretching vibration of the C–N bond in the aromatic ring is detected at 1296 cm^–1^. The absorption band at 1236 cm^–1^ represents the stretching vibration of the C–N^+^ bond of primary aromatic amines in the conductive polaron structure of the polymer. The peak observed at 1110 cm^–1^ arises from the −NH^+^= vibration, and it is associated with charged polymer units Q=NH^+^-B or B-NH^•+^-B.^38^ The region 560–820 cm^–1^ is assigned to the C–H out-of-plane vibrations of 1,2- and 1,4-disubstituted benzene.^39^

The doped conductive state of the elaborated polymer is confirmed by the presence of the peak at 1050 cm^–1^ attributed to the stretching mode of the S=O doping anion.^36^ The emergence of all of these characteristic bands of the oxidized PAni coating confirms that the polymerization of Ani on CFs results in the formation of an emeraldine salt, which is the only conductive form of PAni. In the case of PEDOT, the spectra obtained show several bands at 1350, 1423, 1551, and 1639 cm^–1^, which are assignable to the C=C and C–C stretching modes of the thiophene ring.^40^ Three other bands at 1029, 1159, and 1253 cm^–1^ are attributed to the stretching vibrations of the ethylenedioxy group.^41^ The peaks observed at 666, 838, and 985 cm^–1^ result from the C–S vibrations in the thiophene nucleus.^42^ No band associated with the doping species is detected in this case because the counterion involved in the doping process is Cl^–^.

**Figure 5:**
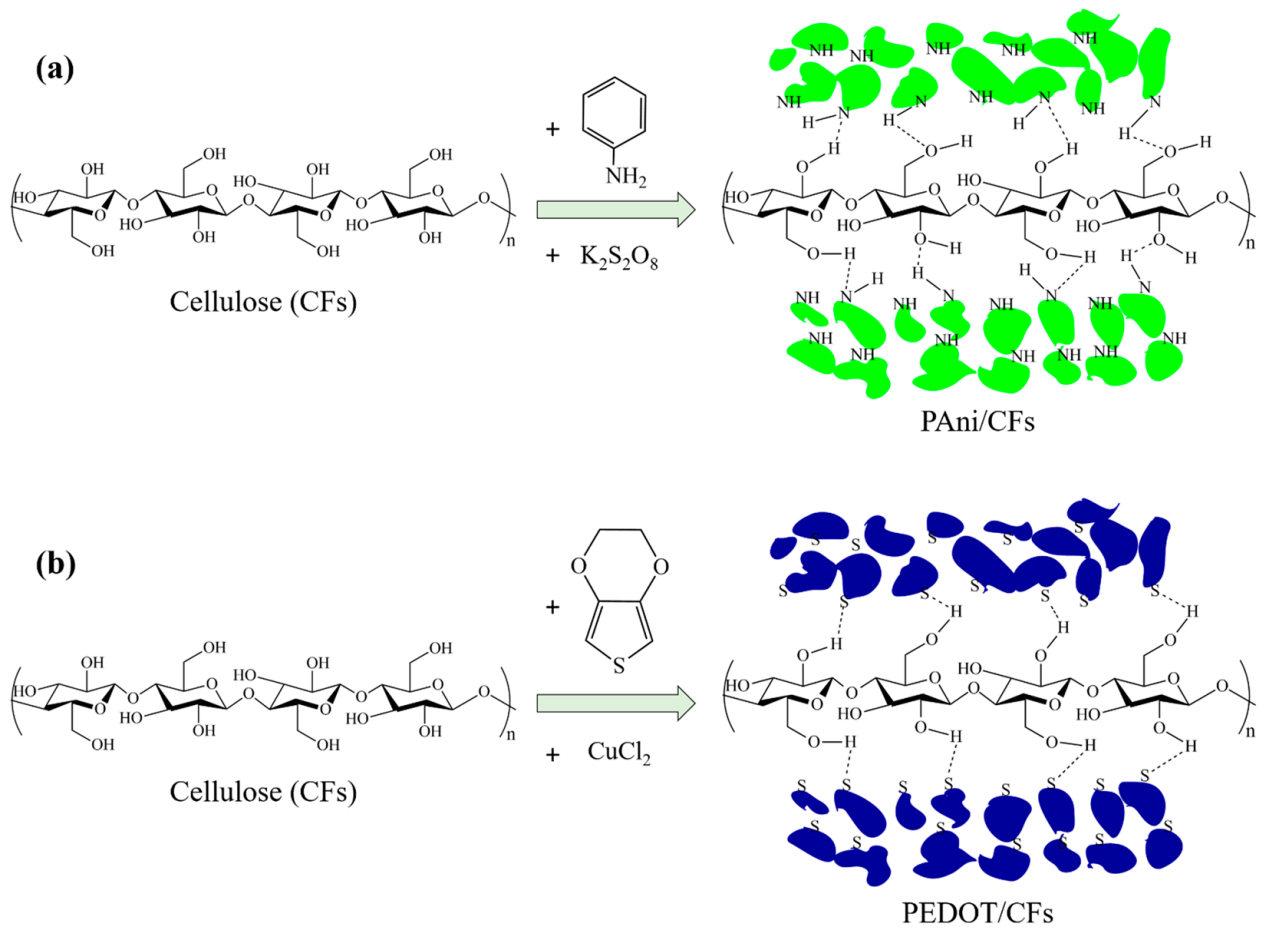
Suggested mechanism of interaction between the CFs and CP, leading to the formation of (a) PAni/CFs and (b) PEDOT/CFs systems.

It should be noted that several bands related to the cellulosic skeleton appear in the spectra of the elaborated coatings, except for the PAni composite, where the CF peaks are not easily distinguished. This observation is probably due to the fact that, in the latter case, the polymeric coating is compact and thick and covers the entire CF. Further justification of this hypothesis will be discussed in the morphological analysis section. Moreover, all major peaks of CFs (particularly O–H vibrations at 3363 cm^–1^) were shifted for the coated fibers, indicating the existence of an interaction between PAni or PEDOT and cellulose moieties. On the basis the previous work concerning PPy,^15^ the formation of a hydrogen bond between the polymer and cellulose could be suggested (Figure 5). This explains the high adherence of the polymers to the cellulosic fibers.

#### 3.1.3 Surface Microstructure

**Figure 6:**
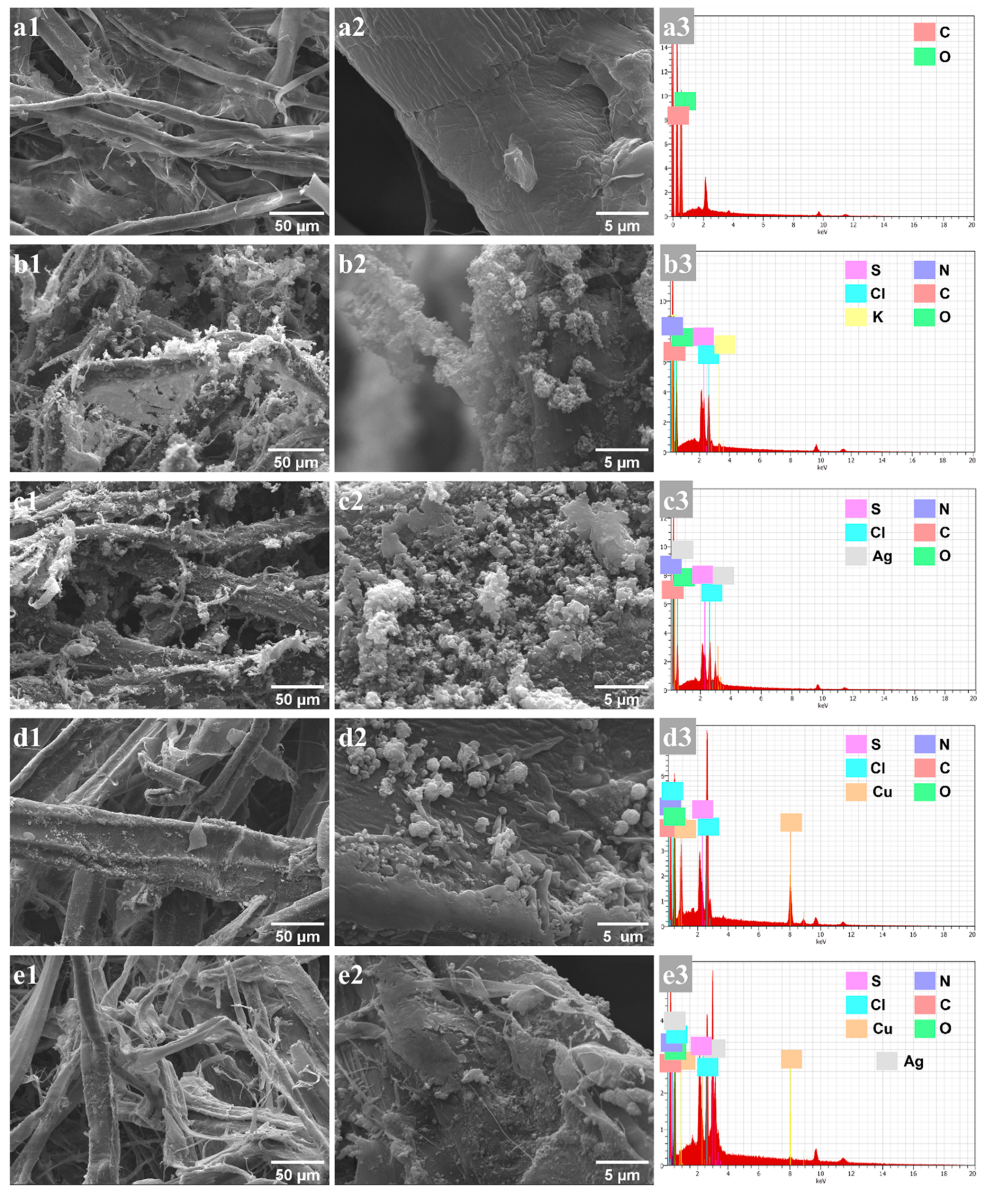
SEM micrographs and corresponding EDS spectra of bare (a1–a3) CFs, (b1–b3) PAni/CFs, (c1–c3) PAni-Ag/CFs, (d1–d3) PEDOT/CFs, and (e1–e3) PEDOT-Ag/CFs.

Additional justification for the successful modification of CFs was revealed from morphological characterizations along with elemental analysis. SEM micrographs before and after the chemical elaboration of CP-based coatings on CFs are presented in Figure 6. It appears that the cellulose-based material has a heterogeneous fibrous structure, with fiber diameters ranging between 10 and 20 μm (Figure 6a1,a2). As expected, the EDS spectrum revealed the presence of only carbon and oxygen atoms related to the cellulosic network of the paperboard used (Figure 6a3). After *in situ* chemical synthesis, the recorded micrographs demonstrated modification of the cellulose surface. For all coatings, analysis demonstrated the presence of aggregated microspherical grains characterizing the polymer. Further analysis of these micrographs revealed the dense network obtained for PAni (Figure 6b,c) in comparison with PEDOT (Figure 6d,e). Indeed, PAni was densely grafted onto the surface, while PEDOT showed a thin coating layer that covered the entire fiber, with very small aggregates distributed along its length. This is in good agreement with FTIR analysis. In both cases, the deposit can sustain repetitive washings and mechanical constraints and remains attached to the fibers.

For PAni coating, EDS analysis reveals the presence of carbon, oxygen, and nitrogen atoms resulting from cellulose and PAni and sulfur and potassium atoms from the doping agent K_2_S_2_O_8_ (Figure 6b3). In the case of PEDOT, apart from carbon, oxygen, and sulfur atoms, we note the presence of copper and chlorine arising from the oxidant CuCl_2_ (Figure 6d3). These results confirm the integration of two polymers at an oxidized conductive state into the cellulose matrix. After the reduction of silver cations, elemental analysis confirmed the presence of AgNPs with atomic percentages of 6.24% and 3.95% for PAni-Ag/CFs (Figure 6c3) and PEDOT-Ag/CFs (Figure 6e3), respectively. SEM–EDS analysis is in total agreement with those of XRD and FTIR.

### 3.2 Gas-Sensing Characteristics

**Figure 7:**
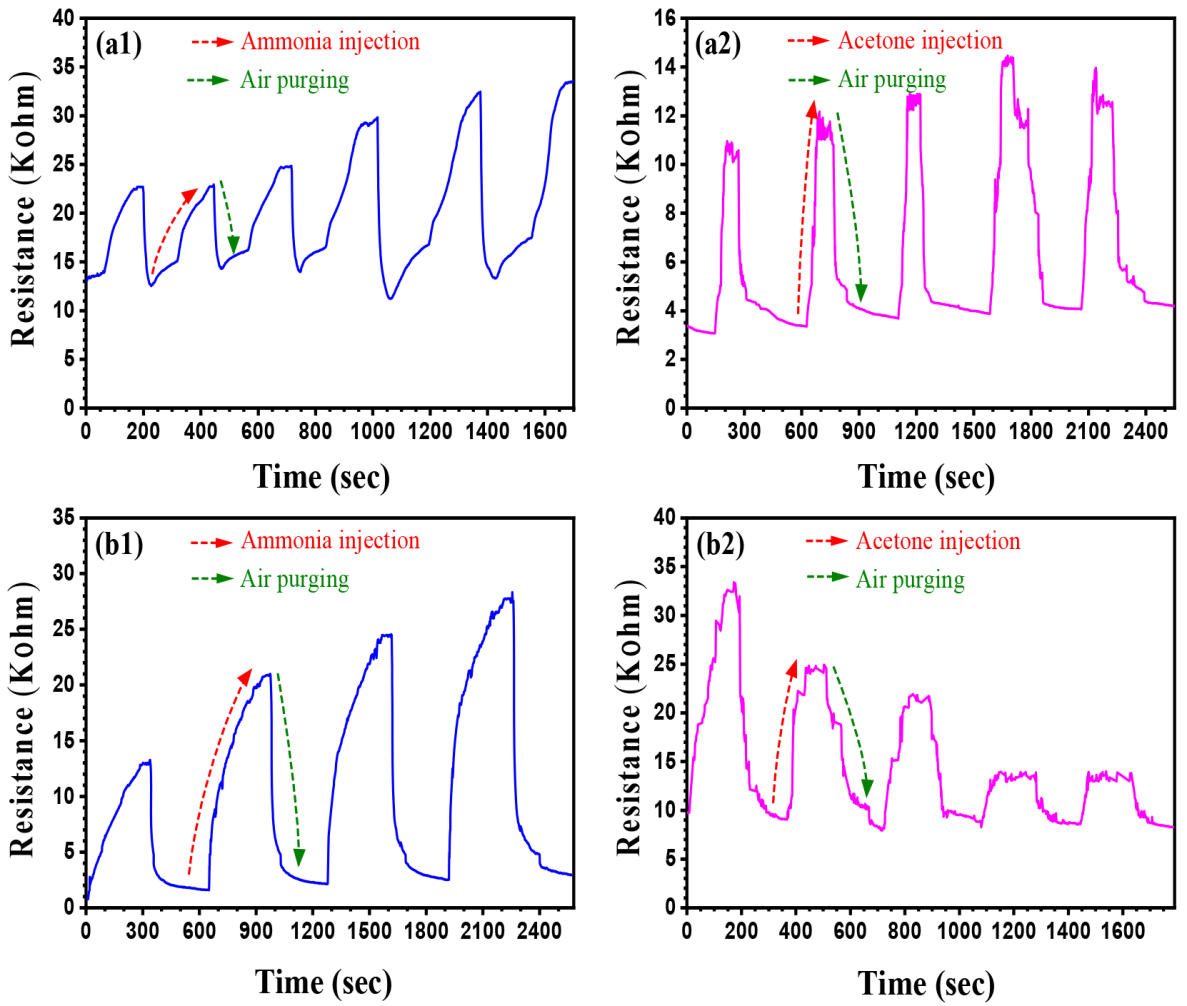
Response–recovery curves of PAni-Ag/CFs (a1 and a2) and PEDOT-Ag/CFs (b1 and b2) upon exposure to toxic vapors.

The present study aims to describe the advanced properties offered by the newly synthesized nanocomposites in order to perform an industrial application in the food-packaging sector. Hence, the elaborated PAni-Ag/CFs and PEDOT-Ag/CFs nanocomposites were first tested for their capacity to detect toxic VOCs. The latter are generally emitted by spoiled food and constitute a real threat to human health. The response–recovery curves of both composites, when exposed to ammonia and acetone vapors, are presented in Figure 7. First, the internal resistance of PAni-Ag/CFs and PEDOT-Ag/CFs before exposition to the gas (*t* = 0 s) is very low (<15 kΩ), which indicates the high conductivity of the coated CFs. In order to strengthen this discussion, simple and low-cost conductivity experiments were conducted based on a homemade assembly in which the polymer was sandwiched between two copper plates. Its impedance (*R*) was then measured using an impedance meter, and its conductivity was deduced by eq 6
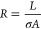
6where *R* is the electrical resistance, *L* is the length, *A* is the area, and σ is the conductivity. The conductivity is found to be 2.1 and 0.81 S/cm for PAni-Ag/CFs and PEDOT-Ag/CFs, respectively. Upon exposure to the vapor, a drastic increase in the resistance is observed in all cases. After air is purged, the resistance of the sensors takes back its initial value. This process is valid for several cycles (gas in–gas out), thus demonstrating the reversibility of the tested sensors. After ammonia exposure, the resistance of the composites increases during successive cycles and reaches values of 34 and 28 kΩ for PAni-Ag/CFs (Figure 7a1) and PEDOT-Ag/CFs (Figure 7b1), respectively. In the case of acetone, saturation of the sensor is detected at a resistance of approximately 14 kΩ for PAni-Ag/CFs (Figure 7a2), while the PEDOT-based coating exhibits a maximum of 33 kΩ in the first cycle (Figure 7b2). This value decreases after the fourth cycle to reach a steady state at *R* lower than 15 kΩ.

Upon exposure to acetone, some molecules of the gas are trapped in the polymeric matrix. Unlike ammonia, it is relatively difficult for acetone molecules to be easily desorbed from the polymer segments when the sensor is exposed to air because of the high molecular weight. Thus, when the number of cycles is increased, the organic-based sensor is partially saturated with adsorbed molecules of acetone. With this saturation, the response of the sensor becomes relatively weak, and the resistance can gradually decline. Despite this, the composite can always detect the toxic vapor, and an instant change in its internal resistance is observed at all cycles. It could be concluded that the sensors are more sensitive to the presence of NH_3_ vapors probably because of the difference in the molecular structure between ammonia and acetone. With the low polarity and high molecular weight of the latter compared to ammonia, it can be difficult for the gas to diffuse through the composite network, which may result in a lower sensing behavior toward this VOC.^43^

The mechanism of interaction has been previously demonstrated in detail with regard to how chemically^15^ and electrochemically^24^ synthesized CPs interact with VOCs. As is known, CPs are p-type semiconductors constituted of neutral and oxidized parts. After ammonia exposure, electronic or protonic exchange occurs between the vapor and oxidized polymeric units. In the case of electron transfer, NH_3_ can provide electrons to the polymer chains, thus transforming the oxidized segments to a neutral state. The second case involves a deprotonation process in which ammonia can capture protons from the backbone of the doped polymer and leads to a decrease in the oxidation rate of the polymer by releasing the doping anions. In both cases, there is a decrease in the conductivity of PAni or PEDOT upon exposure to the vapors, which can explain the increase in the electrical resistance. On the other hand, the interaction of acetone vapor with CPs can manifest through the establishment of reversible hydrogen bonds. Formation of the O–H bond can increase the disorder in the polymer chains and hinder electronic movement, which increases the internal resistance of the CP.

**Figure 8:**
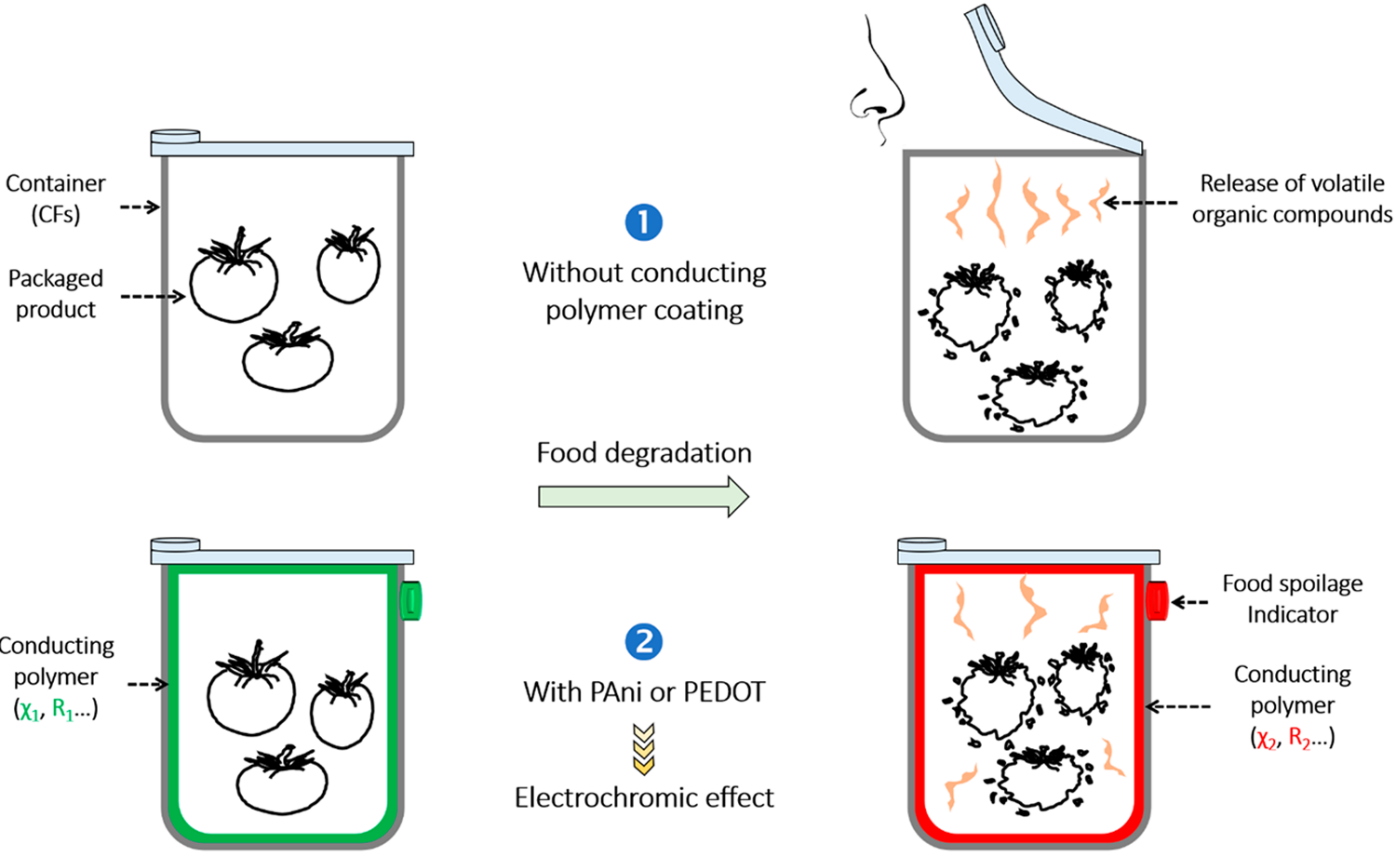
Interaction between toxic vapors resulted from altered food and the outside environment: (1) classical food packaging; (2) smart food packaging.

In summary, CPs–silver/CFs nanocomposites have demonstrated interesting detection properties toward ammonia and acetone. The latter are usually emitted in the surrounding environment when the foods are altered. In the presence of common food-packaging materials, the consumer is subjected to VOCs when opening a package containing altered foods, which may present a big concern to human health [Figure 8(1)], while in the case of CP-based packaging, food spoilage can be detected before opening the container by a color change of the polymer. Indeed, CPs are known for their electrochromic properties. Because of multiple bonds, CPs have absorption bands in the visible and near-UV region, resulting from the presence of π electrons and charged polaron and bipolaron defects. After ammonia or acetone exposure, there is a variation in the oxidation state of the polymer resulting from a change in its internal resistance. This modification in the physicochemical parameters of the polymer (χ, *R*, ...) is accompanied by a reversible change in its optical properties [electrochromic effect; Figure 8(2)].

Generally, the redox behavior of some materials like CPs is used in electronic noses to identify odors like in the olfactive human system. Before such technology, the detection of vapor was achieved by gas chromatography–mass spectrometry, which is considered to be an expansive method with a high response time. With the discovery of CPs, promising sensing applications have recently emerged because of the low-cost production, simple synthesis method, and response time of a few seconds for such materials. Thus, we are in the presence of smart packaging that can be used for the quick and accurate detection of altered food.

### 3.3 Bactericidal Performance

One of the main scientific interests of the proposed topic is to develop antimicrobial coatings based on CPs, capable of providing permanent sterilization of the packaging on which they are deposited. Thus, the antibacterial activity of the coated and uncoated CFs was evaluated on two sensitive strains, *E. coli* and *S. aureus*, using the agar diffusion test. The data pertaining to the antibacterial potential are depicted in Table 1. Concerning bare cellulose, which was used as a control, the results demonstrated a zone of inhibition of 10 and 9 mm for *E. coli* and *S. aureus*, respectively. The two tested strains were considered to be resistant because it was noted that the existence of bacterial growth in the inhibition zone indicates nonantibacterial activity of the uncoated CFs. In contrast, the synthesized nanocomposites are endowed with an important antibacterial activity that is illustrated by an inhibition zone higher than 9 mm. As reported in Table 1, PAni- and PEDOT-coated CFs show good antibacterial activity ranging from 9 to 12 mm. As mentioned in the literature, the CPs are known for their ability to inhibit the microorganisms’ growth as a result of the positive charges present in the polymer skeleton^44^ as well as the negative charge of the doping counterion.^45^

**Table 1:** Inhibition Zones of the Coated and Uncoated CFs

	diameter of the inhibition zone (mm)	
sample	*E. coli* ATCC 25922	*S. aureus* ATCC 29213
bare CFs	10 ± 0.20^a^	9 ± 0.30^a^
PAni/CFs	9 ± 0.21	11 ± 0.20
PAni-Ag/CFs	15 ± 0.13	11 ± 0.15
PEDOT/CFs	11 ± 0.23	12 ± 0.11
PEDOT-Ag/CFs	15 ± 0.15	11 ± 0.18

a Growth in the inhibition zone should be reported as resistant.

In the present study, the inhibition mechanism of the coated CFs involves an electrostatic interaction between the charged nitrogen, sulfur, chloride or also sulfate ions and the membrane cell of the bacteria, which could significantly affect the function and growth of the bacterial cell.^46^ Regarding the composites, PAni-Ag/CFs and PEDOT-Ag/CFs formed by a reduction of silver cations have exhibited a higher inhibition zone against Gram-negative *E. coli* (15 mm for both composites), while the tests achieved for Gram-positive bacteria have revealed no significant change compared to the coating without AgNPs. The synthesized polymer–silver/CFs is more sensitive to the presence of Gram-negative strains. Similar results were obtained in one of the previous works^15^ and by several other researchers in the case of coated cotton fabric^16^ and AgNP-decorated PPy nanotubes.^47^

**Figure 9:**
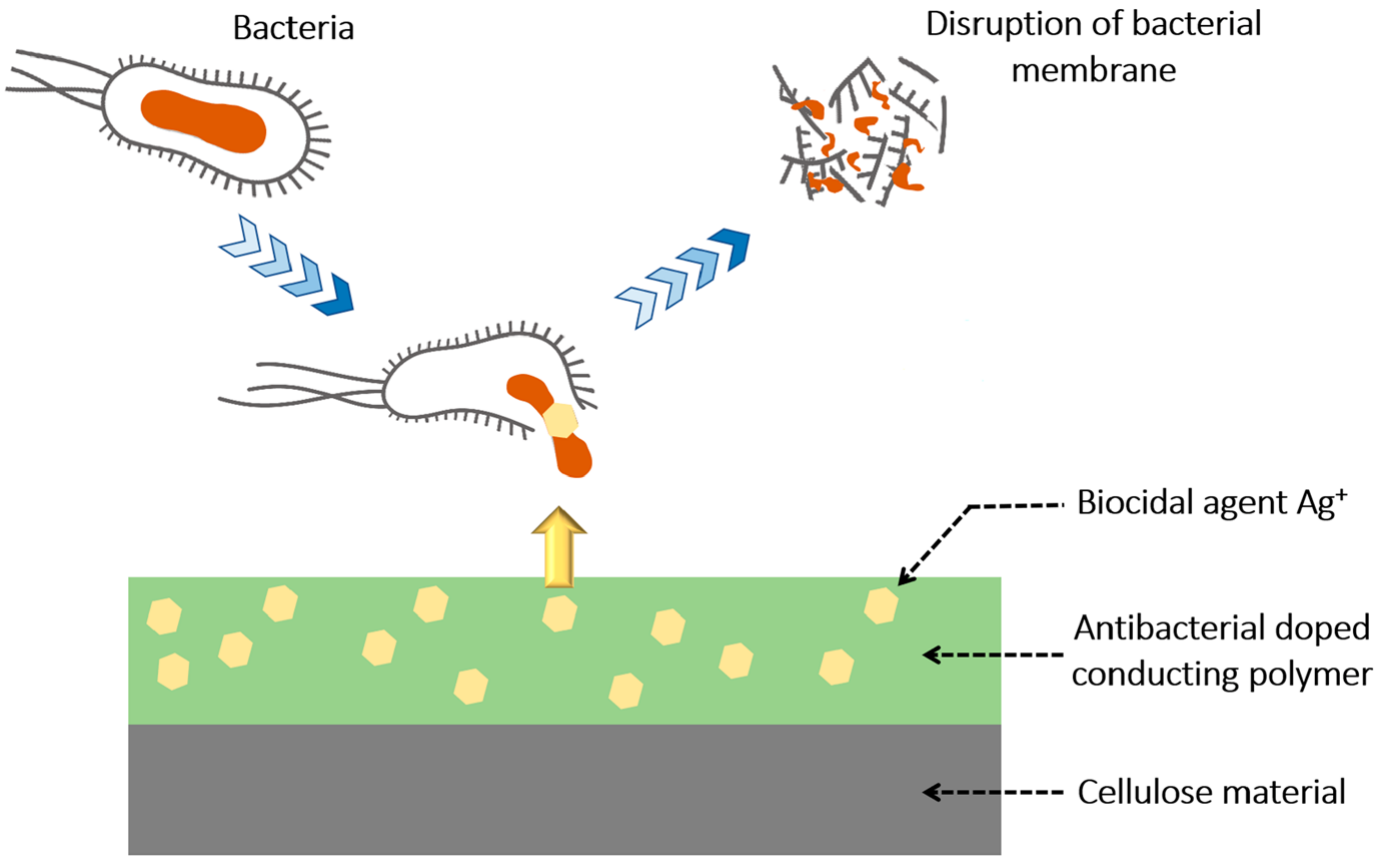
Schematic illustration summarizing the interaction between the incorporated AgNPs and bacteria.

The increase of the antibacterial effect in Gram-negative bacteria *E. coli* could mainly be assigned to the silver ions incorporated in the CP-based system (Figure 9). The AgNPs are well-known for their capacity to cause membrane distortion, which could induce its perforation and thus the release of cell organelles in the external environment. Interruption of adenosine triphosphate production is another result of interaction with the respiratory chain. This disruption could result in the formation of reactive oxygen species (ROS),^19^ which could interact directly with the bacterial DNA and prevent its replication, thus inhibiting the multiplication of bacterial cells.^48^

### 3.4 Antioxidant Capacity

Lipids are a well-known food component. Their susceptibility to oxidation is a key source of food spoilage.^49^ In this context and in order to preserve the optimum quality of the packaged foodstuffs, numerous strategies were deployed to reduce food oxidation.^50^ One of the novel methods related to this subject is the use of antioxidant-active packaging. In other words, instead of using classical approaches such as adding antioxidant species directly to food or combining vacuum or modified-atmosphere food containers with high-barrier materials, the concept of active food packaging is adopted because it can offer a continuous release of antioxidants during storage, which allows the life of packaged products to be extended. In the present study, the elaborated nanocomposites were tested for their capability to reduce DPPH^•^. The assessment of the scavenging activity of this stable free radical is a simple protocol that can allow us to demonstrate the active properties of the prepared samples.

**Figure 10:**
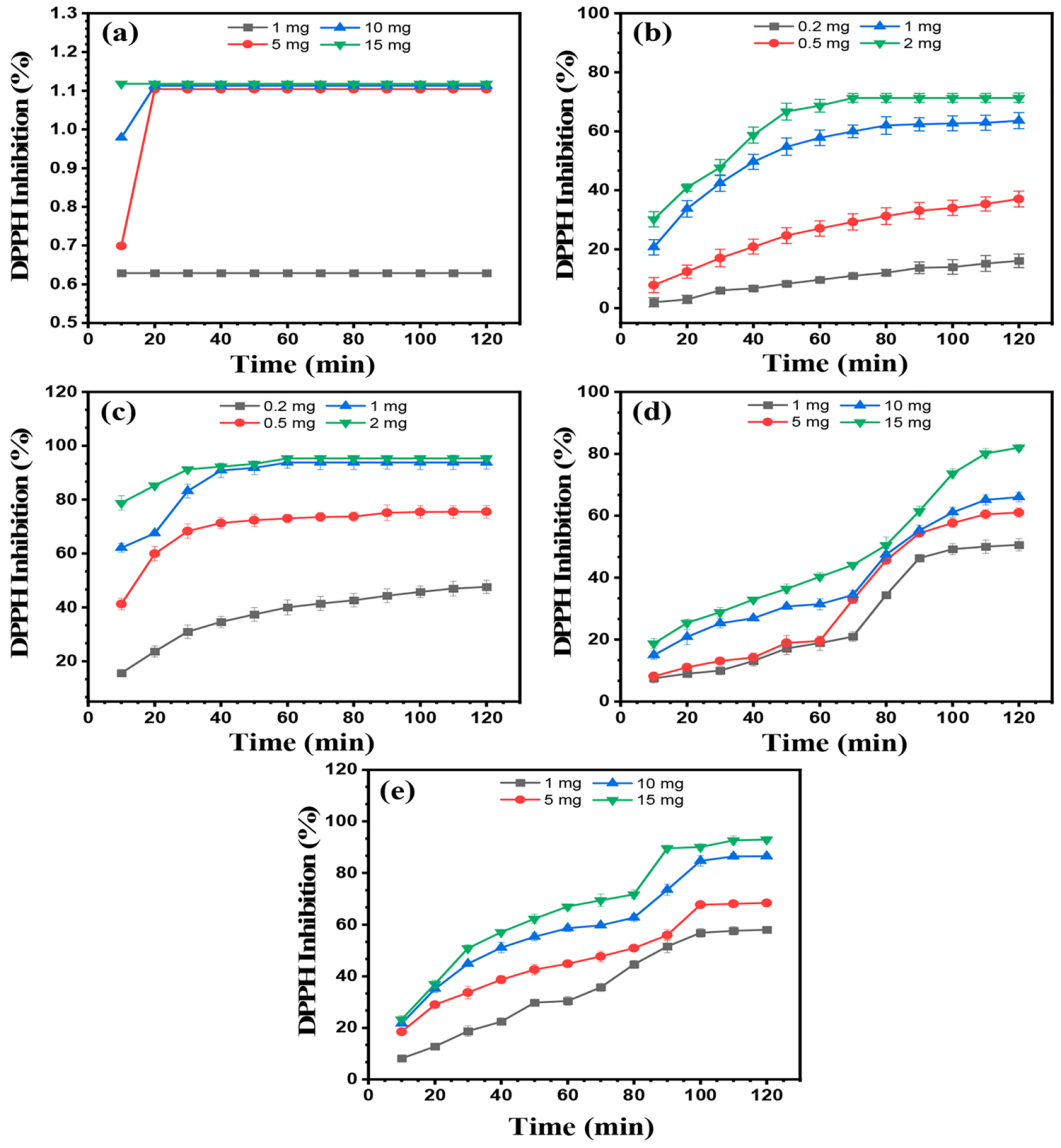
Time-dependent antioxidant property of the coated and uncoated CFs with different contents: (a) bare CFs; (b) PAni/CFs; (c) PAni-Ag/CFs; (d) PEDOT/CFs; (e) PEDOT-Ag/CFs.

The results for the reaction of the DPPH^•^ free radical with various amounts of elaborated materials are presented in Figure 10. For comparison, bare cellulose was also tested, and the percentage of activity is drafted in the same Figure 10. As shown, uncoated CFs reveal weak antioxidant activity with DPPH inhibition of less than 2% even with an amount of 15 mg. The tests realized for the coated samples have demonstrated a progressive increase in the inhibition percentage during an increase of the contact time. In the case of PAni and PEDOT without AgNPs, the results indicate the growth of the free-radical scavenging capability with increasing content of the polymer. DPPH inhibition at 120 min reaches values of about 71.32 ± 1.59% and 81.98 ± 1.01% for 2 mg of PAni and 15 mg of PEDOT, respectively. As is known, antioxidant activity is the capability of a compound to provide active hydrogen atoms or transfer electrons to reduce DPPH^•^ radicals.

Hence, because of its redox-active characteristics, PAni is expected to have strong and efficient properties to reduce DPPH^•^ radicals, while the PEDOT is expected to be less active. As reported in the literature, PAni has the strongest antiradical scavenging activity, followed by PPy and finally PEDOT, which is consistent with the present study findings in which PAni showed better antioxidant capacity compared to PEDOT.^51^ It was also suggested that the PAni antiradical potential could be due to the −N–H group present in the molecular structure, which can donate hydrogen protons that will easily help in neutralization of the stable radical DPPH^•^.^52^ Generally, for neutralization of one DPPH^•^ radical, two Ani units are needed. On the other hand, the low activity observed in PEDOT could be attributed to the lack of heteroatom, with the hydrogen attached accessible for transfer, and to the steric hindrance of PEDOT,^53^ which means that to neutralize one DPPH^•^ radical, eight EDOT units are needed.^51^

Moreover, an important increase in the antioxidant behavior of the composites occurs with the addition of AgNPs. After only 10 min, the PAni-Ag/CFs interact quickly with DPPH^•^, exhibiting a high reduction of 78.77 ± 2.71% for a 2 mg amount. The scavenging activity reaches a maximum of 95.33 ± 1.01% after 60 min of interaction. Similarly, about 92.95 ± 1.66% of the DPPH used was consumed within 100 min by 15 mg of PEDOT-Ag/CFs nanocomposites. The obtained results were in accordance with Hajji et al.,^54^ where it was reported that AgNPs could enhance the scavenging activity of a composite by accepting or donating electrons. Nunes et al.^55^ have assigned the improved antioxidant capability of chitosan–silver composites to bioreductive groups present on the surfaces of AgNPs. From these data, we can conclude that there is a synergistic effect between AgNPs and CPs (PAni or PEDOT), which enhances the antioxidant properties of the nanocomposite.

In summary, the antioxidant capacity is considered to be an important property of active packaging because it helps to increase the shelf-life of food products by absorbing oxidizing agents. The results showed notable scavenging activity of the CPs–silver nanocomposites. Furthermore, these results were found to be more efficient than those reported in previous studies. Indeed, Bideau et al.^17^ have demonstrated that the antioxidant activity of 5 mg of PPy-coated TEMPO-oxidized nanofibrillated cellulose (TOCN) does not exceed 70% for 5 days of contact, whereas in the work of Hsu et al.,^51^ practically no scavenging activity was shown over the first 30 min. However, the activity increases over time and reaches values of 75, 82, and 41% for 1 mg of PPy, PAni, and PEDOT, respectively.

## 4 Challenges and Future Perspectives

In recent years, nanotechnology has proven to be very promising in the food industry. Owing to their promising features, nanomaterials have gained considerable interest from researchers and have considerably contributed to the revolution in the field of biotechnology. They can play a key role in enhancing the barrier properties, stability, and strength of classical food containers and provide a potential antimicrobial activity that makes them highly applicable in the packaging, biomedical, and agriculture fields. In this context, the present study has proven the active/intelligent characteristics offered by AgNPs embedded within a CP-based nanocomposite. This next-generation packaging with multifunctional properties such as antioxidant, antibacterial, and toxic gas sensors can offer optimum protection of the packaged products, which results in a longer shelf-life. Despite the high efficiency of the developed nanocomposites, there are still some issues that need to be addressed in the future. The challenges and perspectives are as follows:

### 4.1 Toxicological Aspects and Safety of Human Health

Because the field of nanoscience and nanomaterials is growing, the potential risk to human health is also progressing. There is public concern regarding the toxicity of active packaging based on nanomaterials and their environmental impact on the consumer. Furthermore, the risk assessment of nanomaterials in food needs to be discussed based on three important considerations: *in vitro* and *in vivo* assays and migration phenomena. Data from the literature show that the morphological and physicochemical characteristics of NPs, including the size and shape, distribution, surface chemistry, and synthesis procedure, among others, play a crucial role in their capability to interact with biological systems and, consequently, influence their toxicity.^56,57^ Despite extensive investigations into their interaction with such systems, the behavior of NPs inside the cells remains a mystery. Several reports have been dedicated to the *in vitro* and *in vivo* analysis of AgNPs toxicity; however, the literature findings are contradictory because of the experimental design and methodology employed in such studies. There is limited agreement and clarity regarding the metabolic pathways affected and the possible deleterious effect on living things.

The toxicity of a cellulose nanofibrils/silver nanoparticles (CNFs/AgNPs) composite to human colon cells (Caco-2 and FHC cell lines) was investigated by Yu et al.^58^ On the basis of the MTT and WST-8 tests, no discernible reduction in the number of viable cells was noted when the addition of the composite was up to 1000 μg/mL. Thus, CNFs coated with AgNPs did not affect human colon cells in any way. In addition, some investigations have shown no genotoxic effect of AgNPs (6–80 nm in diameter) on diverse cell types over 10 mg/mL doses.^59−61^ Other research corroborates the genotoxic potential of AgNPs, with an average size of 1.5–70 nm for human cells.^62−64^ AgNPs of 5 and 28 nm sizes were used to study cell reaction, and Yang et al.^65^ found that the 5 nm AgNPs were more cytotoxic than the 28 nm ones as a result of the high production of H_2_O_2_ in the process. Generally, the toxicity of NPs results mainly from their persistent, nondissolvable, and nondegradable nature. The most important factor of the cytotoxic and genotoxic effects of AgNPs originates from silver ions.^57,66^ If increased above certain levels, the oxidation of AgNPs generates the formation of a silver cation that impair the mitochondrial function caused by oxidative stress, increases the generation of free radicals and other biologically hazardous ROS, and causes cell death, one of the most significant associated toxicity pathways.

On the other hand, one of the limitations of using nanomaterials in the packaging sector is the migration phenomenon, which is described as the transformation of NPs through the container onto the packed products. Such a phenomenon is a result of numerous factors, e.g., temperature, size, and shape of NPs, food condition, polymer properties, and contact time. In the case of smart active systems such as the one developed in the present work, monitoring of the migration and its effect on the food needs to be achieved in all steps of manufacturing using sophisticated analysis like inductively coupled plasma mass spectrometry (ICP-MS). Bideau et al.^26^ have demonstrated that packaging based on CP is safe with regard to the possible particle leaching problems. The authors have shown that only 0.2 × 10^–3^ mol/L PPy was released from the TOCN/PPy composite, which makes the CP-based materials potential candidates for direct contact with food.

A critical review was achieved by Morais et al. to identify research that assessed AgNP migration in food packaging. Only 2 of the 26 publications showed no evidence of migration. However, the validity of these findings is disputed because every study yields conflicting, contradictory, or dubious conclusions. Because some methodological inconsistencies were found in all of the reviewed studies, it was impossible to draw the conclusion that AgNPs found in food packaging have a propensity to migrate to the food matrix, highlighting the need for future investigation. Therefore, in this regard, evaluation of the NPs behavior in various food/simulants and inquiries about the migration capability at various times, temperatures, and types of packaging were performed using the proper techniques.^57^ Such analyses are vital to ensuring that all of the required properties are attained for a food packaging material according to food law compliance regulations.

### 4.2 Food Simulation for Large-Scale Applications

One of the interesting subjects to deal with in the future, besides a study of the toxicity of the composites, is the development of an apparatus in which the newly elaborated materials can be tested as food packaging when placed in contact with foodstuffs. The idea is to design a packaging material based on CPs–silver/CFs that can allow an easy evaluation of the food quality (color, appearance, etc.) over time. In this regard, a piece of food product (banana, cherry tomato, etc.) or a liquidlike milk or juice will be placed inside a flask that is coated on the interior face with the tested nanocomposites. The flask will then be hermetically closed, and the control will be kept in open air at room temperature for several days. With this homemade setup, several experiments can be conducted. It can be found that possible leaching of molecules and NPs embedded in the CP matrix was achieved by analyzing the used packaging products using several techniques, e.g., ICP-MS, total organic carbon, and UV–vis. Additionally, by controlling the texture and appearance of the food, a general idea can be obtained about the antioxidant properties of the tested composite, which can delay the alteration of food.

On the other hand, the smart sensory characteristics of CPs can also be investigated with such a setup. Indeed, by subjecting packaged food to different environmental factors, an assumption of the alteration of the food can be discussed based on the change of color of the CPs. This change can be easily detected because the designed apparatus can ensure the visibility of the polymer that makes the control of any disturbance of the packing system highly flexible.

It should be noted that a simulation in contact with banana and tomato was previously achieved by Bideau et al.^1,17^ for PPy-coated nanocellulose. The authors demonstrated that all foods kept a normal texture with an attractive look for consumption. No brown color was noticeable, which indicates the good barrier property against oxygen and the high antioxidant capacity of CP that can delay food degradation. Thus, by using CP coatings, it is possible to prepare more efficient food containers with improved food preservation properties.

In summary, the packaging sector is anticipated to be significantly impacted by nanotechnology. Moreover, with limited studies on the effect of NPs on human health, the efficiency of such materials as food containers needs further exploration. Thus, with the aim of providing ecofriendly packaging that takes into consideration the safety of the consumer, outstanding topics of studies need to be addressed and include mainly (1) investigation of the toxicity of CPs–silver nanocomposites, (2) their use in real conditions (in contact with food products) to match industrial needs, and (3) evaluation of the migration, toxicity, and biodegradability of CPs–silver in order to demonstrate the biosafety of such composites. These studies will play a major role in the future to boost the possible application of CPs combined with metallic NPs as promising bioactive nanocomposites in food industries and to generate more focus on such a field of research by the scientific community.

## 5 Conclusion

The present work fits into the framework of efforts deployed to discover safe and effective alternatives to classical food packaging. In this context, the use of electronic CPs combined with cellulose as smart contact materials has been demonstrated to be very promising. Homogeneous and strongly adherent PAni and PEDOT with incorporated AgNPs on CFs were elaborated on. The elaborated nanocomposites have shown interesting sensory characteristics toward toxic ammonia and acetone vapors. In addition, PAni and PEDOT exhibited a noticeable antibacterial effect against *E. coli* and *S. aureus* strains, and as expected, the bactericidal performance improved after the incorporation of AgNPs. Moreover, the composites displayed enhanced antioxidant capacity, which improved the shelf-life of perishable packaged products by retarding the oxidation reactions of food components. Packaging industries require the use of advanced materials capable of interacting with their environment and detecting any disturbance in the system for which they are designed. Thus, the new elaborated material could constitute an attractive substitute for conventional packaging because of its smart properties, which can improve the safety of foods and increase their shelf-life. The newly developed method has proven to be suitable for industrial applications because it has demonstrated to be an economic, simple, and environmental process to produce CP-based materials. Therefore, the presented approach can be used to prepare highly efficient food containers on a large scale.
